# Polymer Nanocomposites for Photocatalytic Degradation and Photoinduced Utilizations of Azo-Dyes

**DOI:** 10.3390/polym13081215

**Published:** 2021-04-09

**Authors:** Emily Z. Wang, Yigui Wang, Dequan Xiao

**Affiliations:** 1 Department of Molecular Medicine, Cornell College of Veterinary Medicine Ithaca, Ithaca, NY 14853, USA; zw63@cornell.edu; 2 Center for Integrative Materials Discovery, Department of Chemistry and Engineering, University of New Haven, West Haven, CT 06515, USA; ywang@newhaven.edu

**Keywords:** photocatalytic degradation, azo-dyes, photo-induced isomerization, self-assembly, polymer nanocomposites

## Abstract

Specially designed polymer nanocomposites can photo-catalytically degrade azo dyes in wastewater and textile effluents, among which TiO_2_-based nanocomposites are outstanding and extensively explored. Other nanocomposites based on natural polymers (i.e., chitosan and kaolin) and the oxides of Al, Au, B, Bi, Fe, Li, and Zr are commonly used. These nanocomposites have better photocatalytic efficiency than pure TiO_2_ through two considerations: (i) reducing the hole/electron recombination rate by stabilizing the excited electron in the conducting band, which can be achieved in TiO_2_-nanocomposites with graphene, graphene oxide, hexagonal boron nitride (h-BN), metal nanoparticles, or doping; (ii) decreasing the band energy of semiconductors by forming nanocomposites between TiO_2_ and other oxides or conducting polymers. Increasing the absorbance efficiency by forming special nanocomposites also increases photocatalytic performance. The photo-induced isomerization is exploited in biological systems, such as artificial muscles, and in technical fields such as memory storage and liquid crystal display. Heteroaryl azo dyes show remarkable shifts in photo-induced isomerization, which can be applied in biological and technical fields in place of azo dyes. The self-assembly methods can be employed to synthesize azo-dye polymer nanocomposites via three types of interactions: electrostatic interactions, London forces or dipole/dipole interactions between azo dyes, and photo alignments.

## 1. Introduction

**Figure 1:**
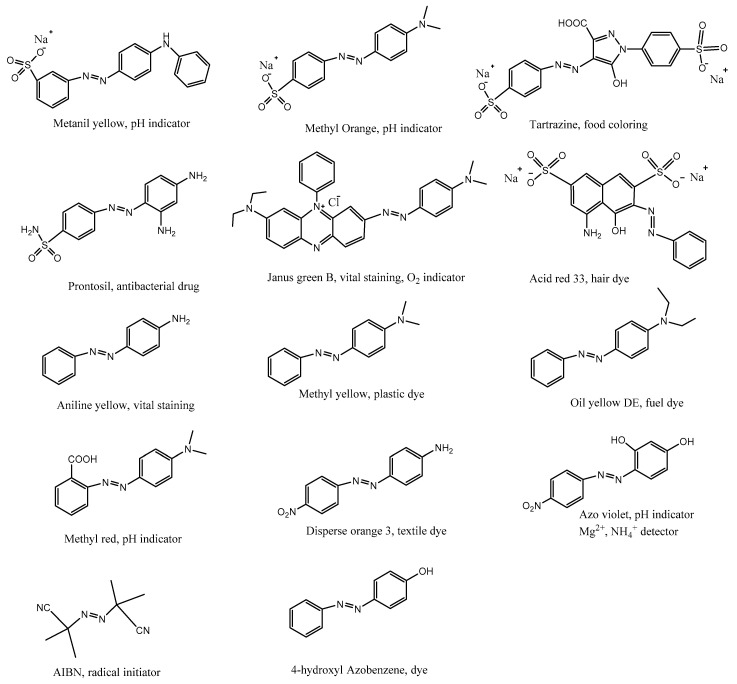
Common azo dyes and their applications.

Azo dyes are dye molecules containing azo (–N = N–) groups. Color-related industries, such as fuel dyes, hair dyes, food dyes, and textile dyes, often use azo compounds [1,2]. Other important scientific fields use azo dyes as indicators for pH measurements, metal ion detection [3], and even O_2_ level monitoring. Azo dyes account for 60–70% of all dyes [4]. Some azo compounds have medical applications. For example, prontosil is an antibacterial drug that was discovered in 1932 after five years of testing thousands of compounds related to azo dyes for useful properties [5]. Figure 1 summarizes representative azo compounds that are commercially available along with their applications.

The outstanding properties of azo dyes invoke extensive applications. The applications of azo dyes in the textile industry naturally release a large number of azo dyes into the environment; thus, the potential hazardous impact of azo dyes on the environment is an imminent threat to the health of human beings [6]. Typical wastewater treatment methods include physical methods (e.g., coagulation–flocculation, adsorption, ultrasound, and membrane filtration), chemical methods (e.g., ozonation, sodium hypochlorite, Fenton reagent (H_2_O_2_–Fe(II)), and photochemical treatment), and biological treatments (e.g., decolorization by fungi, bacterial decolorization, and enzymatic decolorization) [7]. Recent reviews have been available in enzymatic decolorization of azo dyes in wastewater [8,9] and combined treatment approaches for large-scale wastewater treatments [10]. Advanced oxidation processes (AOPs) are highly broad-specificity chemical methods that can quickly convert azo dyes to eco-friendly final products such as CO_2_ and H_2_O. Different combinations of AOPs and other methods top the list of publications, among which photochemical treatment is outstanding. Nanocomposites are photocatalysts in photochemical treatments. In this mini-review, firstly, we will review the development of polymer nanocomposites (most of them contained polymeric materials as a matrix) that are designed for photo-catalytic azo-dye removal in wastewater and textile effluents.

**Figure 2:**
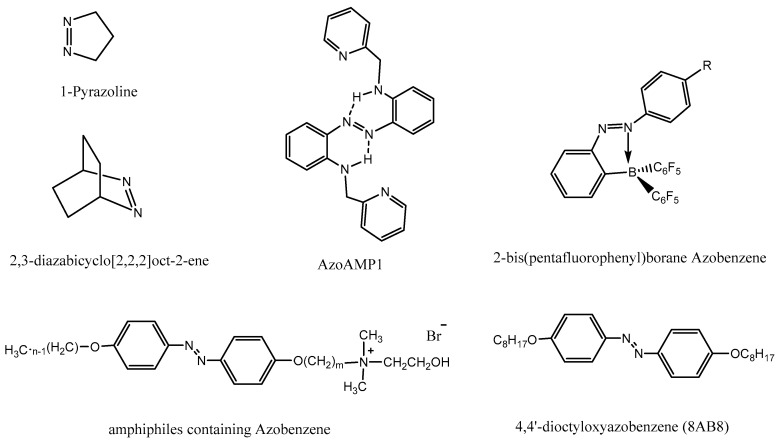
Systems that emit enhanced fluorescence.

Besides the colors, azo-dye compounds display outstanding photo-induced properties. It is well known that cis- and trans-azo-dye compounds can be selectively converted into each other under the irradiation of different wavelengths of lights [11]. The cis-isomer is dominant under UV light while the trans-isomer is the major form in visible light [12]. The phosphorescence of azobenzene has not been reported, because the triplet state is not involved in photoisomerization of azobenzene [13]. However, when the photoisomerization is hindered or blocked, enhanced fluorescence can be observed. Figure 2 summarizes representative azo dyes that emit enhanced fluorescence. Fluorescence has been observed for rigid cyclic azo compounds 1-pyrazoline [14] and 2,3-diazabicyclo(2,2,2)oct-2-ene [15]. The compound 2,2’-bis(N-(2-pyridyl)methyl)diaminoazobenzene (AzoAMP-1) forms strong intramolecular hydrogen bonding that introduces a barrier to photoisomerization, so that AzoAMP-1 adopts a rigid planar structure to be more emissive than azobenzene at low temperatures [16]. Yoshino and co-workers introduced a bis(pentafluorophenyl) borane group to the 2-position of azobenzene (AB). The compounds (R = H, OMe) produce the most intense green fluorescence (at 503 nm and 524 nm) when they are irradiated separately by light at 386 nm and 439 nm. Remarkably, the fluorescence emits at room temperature, and it is the most intense among all azo dyes that have been obtained so far [17]. In this mini-review, we summarize the progress in utilizing the photoinduced properties of azo dyes. We specifically focus on three azo-dye-based materials: photo-control machinery, nonlinear optical materials, and light-aligning crystals.

Polymer nanocomposites are versatile materials that can introduce multiple functions simultaneously, i.e., mechanical strength and catalytic or optical properties. Self-assembly is a facile, effective, and versatile technique that can introduce different azo dyes into polymer nanocomposites. A brief survey on the self-assembly method will be made in this mini-review.

At the end, we provide some promising outlooks for azo-dye-related research.

## 2. Polymer Nanocomposites for Photocatalytic Degradation of Azo-Dyes

### 2.1. Nanocomposite Photocatalysts from Natural Polymers

Chitosan is the most studied natural polymer to form nanocomposites. The SnO_2_/ZnO quantum dot (3–5 nm in diameter) heterojunction immobilized on crosslinked chitosan films (SnO_2_/ZnO/chitosan films) were prepared successfully [18]. The nanocomposite showed good photocatalytic reactivity to remove methyl orange under visible light, and the catalyst was stable and efficient in the reaction conditions. However, chitosan-based nanocomposites are more often used as absorbents [19,20,21,22,23]. Kaolin is another commonly used natural material to form nanocomposites [24,25].

Chitosan and kaolin are exploited by their biodegradable, polycationic, and solid matrix properties in preparing nanocomposites. The photocatalytic properties come from semiconductor components and light irradiation. In other applications, H_2_O_2_, ultrasonic resources, and pH adjustment are required for preparing or applying nanocomposites (Table 1).

### 2.2. TiO_2_ Based Nanocomposites

Many photocatalysts include TiO_2_ (Table 2). Stengl et al. synthesized hexagonal boron nitride (h-BN)–TiO_2_ nanocomposites by thermal hydrolysis of titanium peroxo-complexes in the presence of exfoliated h-BN. The highest rate constants for photocatalytic removal of orange II and reactive black 5 were *k* = 0.0762 min^−1^ and 0.0164 min^−1^ under UV and visible light irradiation, respectively [26]. Sharma et al. obtained surfactant-aided TiO_2_–MgO nanocomposite (Ti–M–S) by sol-gel process with the aid of sodium dodecyl sulfate as structure-directing anionic surfactant. The Ti–M–S efficiently removed methyl orange and methylene blue under visible light irradiation [27]. Karimzadeh and Bahrami [28] used L-cysteine and cadmium sulfide (CdS) as promoted agents to develop new TiO_2_-based nanocomposites and successfully improved the photo-catalytic activity of TiO_2_. Fly ash supported photocatalytic nanocomposite poly(3,4-ethylenedioxythiophene)/TiO_2_ has extended efficiency to remove reactive red 45 under solar light [29].

Doping TiO_2_ has become a popular way to improve the photocatalytic activity of TiO_2_. Zangeneh et al. [30] doped TiO_2_–CuO with N(Urea) and C–N (L-Asparagine) and found that C–N doped TiO_2_–CuO had the highest photocatalytic activity for direct red 16 removal. A novel fixed-bed continuous-flow photoreactor was developed using N–Fe-doped TiO_2_/SiO_2_ and visible light to simultaneously remove Cr(VI) and azo dyes [31]. The Cu doped TiO_2_–Bi_2_O_3_ hybrid had the best photocatalytic activity with 1% Cu and 8% Bi_2_O_3_ in visible light [32]. The Mn, Mo, and La/TiO_2_/activated carbon (AC) exhibited high absorption ability in the visible light region [33], while the Fe^3+^ doped TiO_2_ efficiently removed nonbiodegradable orange II [34].

Nanocomposites of TiO_2_ could be formed in other ways. Heat treatment of ZnO/TiO_2_ transformed the nanocomposite into ZnTiO_3_ and Zn_2_TiO_4_ [35]. The TiO_2_ component has been included in core–shell nanospheres of which the cores were magnetic. For instance, the TiO_2_ that encapsulated ferrites could enhance the removal of azo-dye in textile effluents under both low- and high-energy lamps [36]. The Fe_3_O_4_/SiO_2_/TiO_2_ nanospheres removed 90.2% and 100% of binary methyl orange and methylene blue in aqueous solution under UV light irradiation (365 nm) for 5 h [37]. In addition, TiO_2_ based nanocomposites can be formed as hydrogel. The TiO_2_ component did not affect the already formed covalent bonds within the polymer network in the TiO_2_/hydrogel nanocomposites, but the colloidal TiO_2_ could completely remove color index (C.I.) acid red 18, C.I. acid blue 113, C.I. reactive black 5, and C.I. direct blue 78 [38]. The pH-sensitive TiO_2_-containing hydrogel could remove 94% acid red 88 and 71% acid orange 7 in 30 min under UV light, and the catalysts could be retrieved at the end of the reaction [39]. Furthermore, the uniform TiO_2_–SiO_2_ nanospheres had remarkably enhanced photocatalytic performance [40,41], while the Au nanoparticles (NPs) decorated TiO_2_-formed nanocomposites with Preyssler acid that could efficiently remove malachite green under UV light irradiation [42]. The TiO_2_/Bi_2_O_3_ nanocomposites (from the ultrasonic-assisted synthesis) increased the removal efficiency of orange II to 94.7% (from 44% for pure TiO_2_ nanoparticles) [43]. At last, the ternary graphene/TiO_2_–Ag composites remarkably removed 85.3–98% of amaranth dye in 2 h under UV and solar irradiation [44], and the ZnO/TiO_2_ nanocomposites coated on stainless-steel electrodes are powerful photoelectrocatalysts, because they enhance hydroxyl radical production [45]. Yu and coworkers [46] prepared a tubular titania nanostructure via layer-by-layer self-assembly, and the photocatalytic efficiency of the nanostructure toward the degradation of methyl orange was found to be dependent on the coating thickness.

### 2.3. Photocatalytic Mechanism of TiO_2_ Nanocomposites

**Figure 3:**
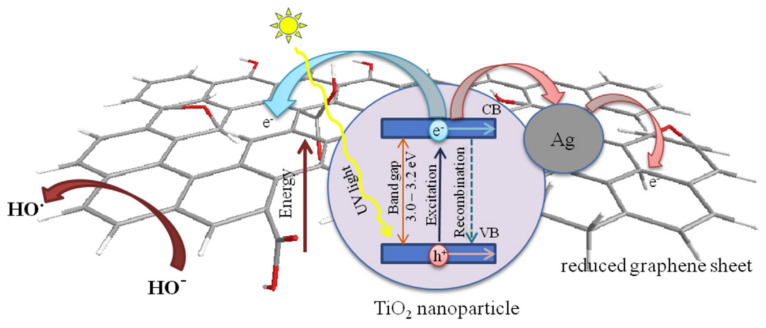
The photocatalytic mechanism of graphene/TiO_2_–Ag nanocomposites [44]. Reproduced with the permission of *Mater. Chem. Phys*.

A typical reaction mechanism is given in Figure 3 for graphene/TiO_2_–Ag nanocomposites to photocatalytic degradation of azo dyes.

Absorption of light excites the electrons from the valence band that are transferred to the conducting band of the semiconductor causing hole/electron (h^+^/e^−^) separation. This results in the reaction of electrons in the conducting band with oxygen to form ·O_2_^−^ superoxide radicals and holes in the valence band with the water to form ·OH radicals responsible for the oxidation process of azo dyes.

O_2_ + e^−^ → ·O_2_^−^ H_2_O + h^+^ → ·OH (1)

The TiO_2_ semiconductors have a large band gap (E_g_ = 3.2 eV) so that only UV light can excite electrons from the valence band (VB) to the conducting band (CB). In addition, TiO_2_ semiconductors have high hole/electron (h^+^/e^−^) recombination rates. Therefore, there are two ways to enhance photocatalytic efficiency, i.e., reducing the energy gap and lowering the h^+^/e^−^ recombination rate. Table 2 summarizes the specific ways to achieve this goal. In the example, the electrons in the conducting band can move to a graphene surface via Ag or a graphene oxide surface; therefore, the hole/electron recommendation on TiO_2_ is eliminated. The hexagonal boron nitride behaviors, such as graphene and doping, is an efficient way to reduce hole/electron recommendation rates. The benefit of reducing the band gap of semiconductors is to utilize the abundant visible lights of solar energy. The band gap reductions are usually achieved through forming nanocomposites between TiO_2_ and other metal oxides or conducting polymers.

### 2.4. Other Nanocomposite Photocatalysts

Other photocatalysts (Table 3) for the degradation of azo dyes were reported in the form of nanocomposites based on Al [47], Au [48], Bi [49,50,51], Fe [52,53,54,55], Li [56], Ni [57], and Zr [58]. The Fe-based photocatalysts are exploited for their easy separation due to the fact of their magnetic properties.

Silver nanoparticles (AgNPs) were functionalized to nanocomposites by utilizing *Portulaca Oleracea* leave (PNL) aqueous extract as a reducing reagent. The nanocomposites are an important part of one-pot green and sustainable approaches for the photodegradation of reactive textile dyes. They also possess antibacterial and antidiabetic potentials [59].

**Figure 4:**
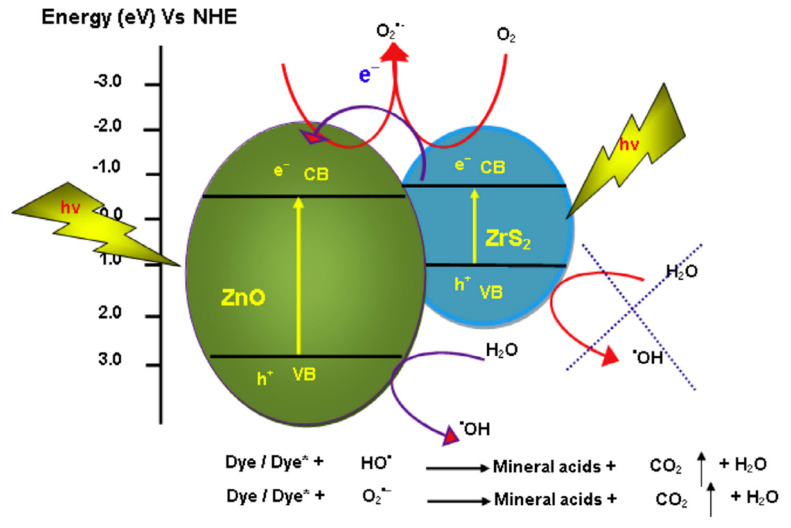
Photocatalytic mechanism for ZnO/ZrS_2_ nanocomposite [58]. Reproduced with the permission of *Separat. Purif. Tech.*

Figure 4 indicates the interplay of two metal oxides in nanocomposites. The strategies of improving photocatalytic performance still lie in reducing the band gap and reducing electron/hole recombination rates. The nanocomposite of two metal oxides can benefit from the reduced band gap due to the heterojunctions. Another way to increase photocatalytic activity is to increase the light absorbance efficiency. One last interesting method is to include magnetic Fe_3_O_4_ for easy separation of the catalysts.

## 3. Polymer Nanocomposites Utilizing Azo Dyes

### 3.1. Photo-Control Machinery

We mentioned the photo-switch properties of azo dyes and enhanced fluorescence through restricting photo-isomerization in Section 1. New exciting applications can be found at molecular, polymer, and nanocomposite levels.

One remarkable application at the molecular level comes from the development of photoswitchable anti-cancer drugs [60]. Recently Trauner and co-workers [61] developed an inhibitor of microtubules called photostatin (PST), which is an azobenzene derivative with four OCH_3_ groups and one ·OH group. Photostatins exposed to blue light are 250 times more cytotoxic than PSTs kept in the dark. Photostatins are “off” in the stable trans-form when kept in the dark and are “on” in the cis-form when put under blue light. Thus, the drugs can be safely applied in large doses globally and only activated locally in cancer tissues by shining blue light.

Azo dyes are an important moiety of shape-shifting polymers that can harvest sunlight energy [62,63]. Heteroaryl azo dyes, which replace one or both benzene rings of azo dyes by heteroaryl rings (such as pyridine, pyrimidine, pyrazole and indazole, imidazole etc.), have been explored as molecular photo-switches. Certain heteroaryl azos can exist in metastable cis-forms for anything from hundreds of picoseconds to more than 1000 days [64]. The chiral optical properties are demonstrated in polypeptides in helix formation and liquid crystal [65]. Among other interesting applications, the polypeptides are found to experience light-induced helix reversal after the azo dyes are bonded to polypeptides as sidechains. A linear polymer with azobenzene as a main chain component can act as a photo-induced motor in which two mechanical steps and two photo-induced steps are required in order for the motor to actuate [66]. Barrett and coworkers reviewed recent developments towards optical control of bio-interfaces. For example, Azo photo-switches could be incorporated into natural polymers thus influencing living systems, and photo-switchable soft azo dye materials could be employed to develop artificial muscles and light-powered soft robotics [67].

Diaz et al. developed biodegradable polymer/azo-dye film by the addition of carbon nanotubes and found that the maximum optical anisotropy was obtained 15 °C below the glass transition temperature. The properties were interpreted as the interactions among disperse orange 3 (OD3), multi-walled carbon nanotubes (MWCNTs), and poly(lactic acid) (PLA) and the packing density of azo dyes into the polymer chain [68]. Georgiev et al. synthesized nanocomposite films of perylene bis azo-imides and showed that the nanocomposites had improved photo-responsiveness so that they can be used as visible-light-activated molecular switches [69]. Zhang et al. prepared a photoisomerizable surface using an azo dye/TiO_2_ nanocomposite film. The azo dye, para-methyl red, undergoes isomerization at room temperature on the TiO_2_ nanoparticle supports. They can be potentially used in solar cells [70].

### 3.2. Nonlinear Optical Materials

Raposo and coworkers synthesized donor-acceptor (DA) thienylpyrrole azo dyes by replacing one benzene ring of azo dye with thienylpyrrole ring, which could potentially be used as nonlinear optical materials in optical communications [71]. Churikov et al. reviewed the third-order nonlinear optical phenomena in azo dye polymers [72].

Liquid crystal photo-alignment of azo dyes was found to be different from previous ones such as photo-crosslinking, photo-degradation, and photo-isomerization. It can provide a controllable pretilt angle and strong anchoring energy of the liquid crystal cell, so it is very promising to use in the liquid crystal display (LCD) industry [73]. The azo dye, methyl red, dopes on the nematic liquid crystal (LC) pentycyanobipenyl (5CB), giving rise to colossal nonlinear or to light-induced anchoring and memory effects, so the methyl-red-doped liquid crystals have a potential for photonic applications [74,75].

### 3.3. Light Aligning Materials

The light aligning properties of azo dyes can be explored together with the self-assembly method to synthesize liquid crystals.

Kumar and coworkers employed in situ self-assembled dual-wavelength photo-alignment to obtain highly stable pre-tilted homeotropic alignment of liquid crystal [76]. Kundu et al. demonstrated a reliable, practical, and cost-effective “one-pot approach” for a homeotropic alignment of nematic liquid crystals that is achieved by photochromic trans- to cis-isomerization of the azo dye doped in a nematic host [77]. Yeung and coworkers described the formation kinetics and related optical properties of a self-assembled monolayer [78].

**Scheme 1:**
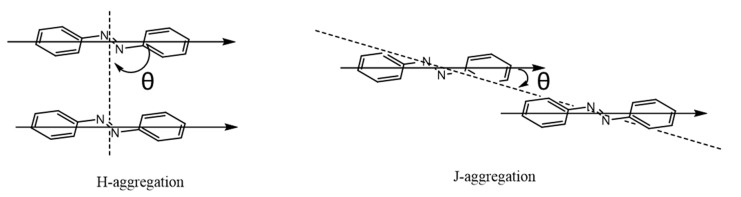
The H- and J-aggregations and related geometrical parameters for a azobenzene dimer.

The common situation for the self-assembly of azobenzene (AB) derivatives is to associate them with polymers to take advantage of the interactions between the phenyl rings of AB derivatives. The interactions between AB derivatives can be London force or dipole/dipole interactions. The aggregation patterns can be J- and H-types that separately represent head-to-tail and one-on-top-of-the-other arrangements. Depending on the angle θ (Scheme 1), the maximum absorption in UV-vis spectra of the assembled AB derivatives demonstrated blue shift or red shift separately for H- and J-aggregations in comparison with free AB derivatives. This phenomenon was clearly explained by the following equation [79]:

Δε = 2M^2^(1 − 3cos^2^θ)/*r*^3^ (2)

whereas M is the dipole moment, *r* is the center-to-center distance between molecules, and θ is defined in Scheme 1.

It is clear that (1) when θ = 54.7°, no splitting (change) occurs in the maximum absorbance peak; (2) when θ > 54.7°, there is a blue-shift; and (3) when θ < 54.7°, there is a red-shift.

Tong and co-workers synthesized the azobenzene-containing diblock copolymers through the atom transfer radical polymerization technique [80]. The percentages of three different aggregation states among azobenzene units (H, J, and free) are estimated at 25 °C and 80 °C. With the reference to the photochemical LC-isotropic phase transition, the authors pictured that the anchoring (ordering) at the interface might slow down the propagation of the perturbation of cis isomers that were responsible for the destabilization of the ordered LC phase.

Menzel and co-workers studied Langmuir–Blodgett (LB) films of poly(5-(2-(4-((4-hexylphenyl)azo)phenoxy)ethyl) L-glutamate) (P2) and poly(5-(6-(4-((4-hexylphenyl)azo)phenoxy)hexyl) L-glutamate) (P6) polymers with a stiff backbone and flexible sidechains (“hairy rods”) [81]. The original LB structure, which is characterized by deformed “hairy rods” arranged in bilayers, is destroyed upon photoisomerization or annealing. The sidechains relax to a more symmetrical distribution around the main chain helix. Furthermore, the aggregation of the chromophores as well as their orientation in respect to the main chain helix is changed. The polymers with different spacers (P2, P4, and P6) confirmed that the supramolecular order of LB films of these thermotropic polypeptides established due to the fact of irradiation and/or annealing is determined by the enthalpic stability of the mesophase and the dynamic properties of the polymers [82].

Bilayer and liquid crystal situations show significantly enhanced fluorescence for azobenzene derivatives. Shimomura and Kunitake reported the strong fluorescence enhancement when the azobenzene-containing amphiphiles were included in aqueous bilayer system, which was explained by head-to-tail J-like aggregation with π–π* characteristics [83]. Tamai et al. reported the fluorescence properties of an azobenzene liquid crystal, 4,4′-dioctyloxyazobenzene (8AB8), as a function of temperature by picosecond single-photon timing spectroscopy. They assigned the fluorescence peak at ~590 nm to the S_1_ fluorescence of a J-like aggregate of 8AB8 and assigned a weaker peak at ~420 nm to π–π* fluorescence [84].

## 4. Self-Assembly Method to Introduce Azo Dyes into Polymer Nanocomposites

The self-assembly method is an efficient way to introduce azo dyes into nanocomposites. Besides the photo-alignment approaches, two major properties are commonly employed: (1) London forces or dipole/dipole interactions between azo dyes; (2) electrostatic interactions for which azobenzene (AB) derivatives could have ionic substituent (or hydrogen bond-forming substituent) or polymers are Zwitterionic polymers.

He et al. prepared NiFe-layered double hydroxide-based azobenzene composite films through the self-assembly method, and the composite films have good stability and a high acid-base gas sensitivity [85]. Zhuang et al. obtained nanoaggregated dispersed red 1-functionalized poly(N-vinylcarbazole) film via solution-phase assembly, and the device showed an ON/OFF current ratio of 10^5^ [86].

Ionic self-assembly (ISA) strategy was used as a supramolecular approach to easily synthesize optically anisotropic materials in which ethyl orange azo dye was complexed with double-tailed ammonium surfactants [87]. The azobenzene containing Zwitterionic polymers can self-assemble into photo- and thermal-responsive polymers that are water soluble and applicable in biological systems [88]. The self-assembly of multilayer diazonium resin, J-acid, photochromic spiroxazine, and poly-(sodium-4-styrene)sulfonate (PSS) can be conducted. The materials can potentially be used in data recording, optical switching, displays, and non-linear optics [89]. Holographic surface relief gratings (SRGs) were fabricated on composite films assembled by electrostatic layer-by-layer (LbL) deposition of a polyelectrolyte, poly(dimethyl diallylammonium chloride) (PDAC), and an azo dye, Congo red (CR). This approach provides a methodology to fabricate SRGs for optical information storage [90].

Polymethacrylate containing azopyridine side chain (PAzPy) forms diblock copolymer with polystyrene, and the azopyridine moiety enabled the easy self-assembly to effectively load zinc–tetraphenylporphyrin (ZnTPP) inside microdomains of PAzPy block [91]. Azo dye chromotrope-2R (CH2R) and polycation (poly(allyamine hydrochloride) (PAH) were successfully deposited onto solid surfaces as a film through layer-by-layer electrostatic self-assembly. In LbL films, the more closure association of dye molecules causes the aggregation of dye molecules, which is reflected in their absorption and steady-state fluorescence emission spectra when compared to those of pure solutions [92]. Hydrogen bonds were employed to synthesize supramolecular azopolymer—a multilayered composite through alternative stacking. The optical birefringence was observed under two-photon excitation [93]. The layer-by-layer self-assembly (LbL) technique was used to obtain poly(p-phenylenevinylene)/Congo red thin film. The LbL PPV/CR film thermally converted at 110 °C and azo dye Congo red experienced a considerable decrease in degradation in the polymer matrix [94]. The electrostatic interactions were used to obtain self-assembled nanoparticles from linear flexible polyelectrolytes and an ionic photo-isomerizable acid yellow 38 (AY-38). The UV light exposure triggers a size change from *R_h_* = 94 nm to *R_h_* = 62 nm for a sample with a charge ratio of *I_charge_* = 0.7 [95].

**Scheme 2:**
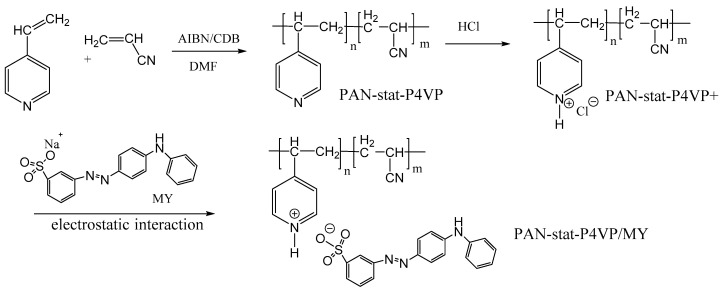
Synthetic routes for poly(acrylonitrile)-stat-poly(4-vinyl-pyridine) (PAN-stat-P4VP) copolymers and PAN-stat-P4VP/MY complexes. Accronyms: AIBN, azobisisobutyronitrile; CDB, cumyl dithiobenzoate; DMF, dimethylformamide; and MY, metanil yellow.

The following is a typical application of self-assembly methods to incorporate azo dye into co-polymers. Jin et al. synthesized the copolymers poly(acrylonitrile)-stat-poly(4-vinyl-pyridine) (PAN-stat-P4VP) from reversible addition-fragmentation chain-transfer (RAFT) polymerization (Scheme 2) [96,97].

**Scheme 3:**
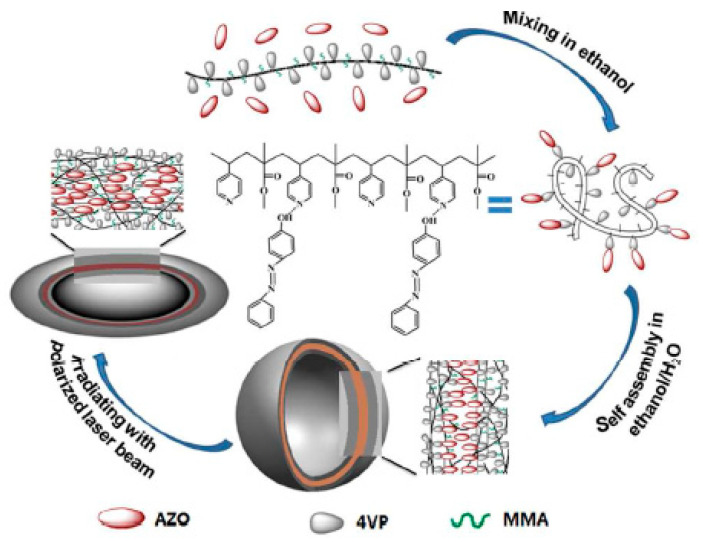
Schematic illustration for the formation process of poly (methyl methacrylate-stat-poly (4-vinylpyridine)) (PMMA-stat-P4VP)/AZO hollow nanospheres and elongated hollow nanospheres [98]. AZO denotes azo dye molecules. Reproduced with the permission of RSC Advance.

The formed PAN-stat-P4VP/MY complexes were identified to be nanospheres using transmission electron microscopy (TEM). The size of the hollow nanospheres was homogeneous and could be conveniently controlled by varying molar ratios of the copolymers to MY and molar ratios of monomers of the copolymers. The formation process is illustrated in Scheme 3.

Using a similar procedure, Jin and coworkers synthesized poly (methyl methacrylate-stat-poly (4-vinylpyridine)) copolymer (PMMA-stat-P4VP). The PMMA-stat-P4VP associates with 4-phenylazophenol by hydrogen bonding and by forming nanospheres. Under the irritation of a linearly polarized Ar^+^ laser beam, the nanospheres underwent a striking deformation of shapes [98,99].

## 5. Conclusions and Outlook

Polymer nanocomposites for azo-dye degradation or removal could be made from a variety of resources such as natural polymers (chitosan, fly ash, and kaolin), synthesized polymers (exfoliated h-BN, hydrogel, etc.), and the oxides of Al, Au, Bi, Fe, Li, Ni, and Zr. The TiO_2_-based nanocomposites are among the most explored and widely used photocatalysts for the degradation of azo dyes in wastewaters and textile effluents. These nanocomposites have better photocatalytic efficiency through two considerations: (1) reducing the hole/electron recombination rate by stabilizing the excited electron in the conducting band, which is achieved by forming TiO_2_ nanocomposites with graphene, graphene oxide, hexagonal boron nitride (h-BN), metal nanoparticles, or by doping TiO_2_-nanocomposites; (2) decreasing the band energy of semiconductors by forming nanocomposites between TiO_2_ and other oxides or conducting polymers. Future research in nanocomposite catalysts may include the followings: (1) developing novel nanostructure photocatalysts that show strong photoabsorption efficiency, which will lead to the increase in photocatalytic efficiency; (2) purposefully introducing polymer materials to reduce or eliminate the electron–hole recombination in nanostructured photocatalysts; (3) using economical and less hazardous nanocomposites catalysts for the degradation of azo dyes, which can avoid further contamination of water.

The photo-induced isomerization has meaningful applications in biological systems, such as artificial muscles, and in technical fields such as memory storage and liquid crystal display. Heteroaryl azo dyes show remarkable shifts in photo-induced isomerization, which might be applied in biological and technical fields in place of azo dyes.

The photoinduced properties are different at molecular, polymer, and nanocomposite levels. An overview from three levels would inspire us to learn from the property changes due to the size and dimension increase and then exploit these properties. For example, the photoisomerization will be hindered when the azo dyes are restrained in polymer materials, but nanocomposites could incorporate azo dyes inside the matrix of supporting materials. Liquid crystal and optical control of bio-interfaces are other examples to properly exploit the azo dye properties. The azo dyes will definitely be outstanding if we properly put them into use together with other compounds. Azo dyes unveil their many faces such as hazards from textile industry, anti-cancer and anti-bacterial drugs, photo-control switches, and memory storage media. More importantly, they lend hands for human beings to interact with the world and harvest solar energies. Azo dyes have been directly incorporated into polymer nanocomposites promising for activated molecular switch, optical recording media, and solar cells. The self-assembly method has shown a promising future in synthesizing polymer nanocomposites containing azo dyes.

Due to the special optical properties of azo-dye molecules, we expect that more novel polymer nanocomposites containing azo dyes will be designed and synthesized to meet the needs of special applications such as optical actuation and controlled drug delivery. Particularly, the self-assembly approach can be applied for the fabrication of these polymer composites. Along this direction, extensive research efforts may be focused on the precise control of the photo-induced properties of azo-dye-based polymer nanocomposites.

## Figures and Tables

**Table 1:** Nanocomposite photocatalysts based on natural materials.

Base	Morphology	Advantages	Mechanisms	Usage	Other Requirements	Stability + Reusability	Reference
Chitosan (CS)	Quantum dot SnO_2_/ZnO (3–5 nm) on chitosan film	Reduced heterojunctions are immobilized on an absorbent matrix; easy separation	·OH and ·O_2_^−^ radicals are active species	Methyl orange (MO) degradation	Visible light	Efficiency only drops after 3 runs	[18]
Chitosan	Chitosan-layered ZrP nanoparticles (NPs)	Synergetic effect (chitosan reduces Cr(VI) then ZrP absorbs Cr(III))	The superoxide anions were stabilized by ZrP or CZrP	Cr(VI), Reactive blue 21 (RB-21), and Reactive red 141 (R-141)	H_2_O_2_	Efficiency unchanged after 3 cycles	[19]
Chitosan	Chitosan-coated CuO NPs	Ecofriendly	Bacteria inhibitor; produces ·OH and superoxide radicals	Bacteria inhibition; Methyl blue (MB) and Congo red (CR) removal	Sunlight		[20]
Chitosan	Chitosan/carbon nanoflowers (CNFs)	Chitosan is biodegradable; carbon nanoflowers act as an absorbent	CS/CNFs have a positively charged surface and can form hydrogen bonds	Acid black 1 (AB-1) and Congo red (CR) removal		50–79% efficiency after 4 cycles (remains)	[21]
Chitosan	Graphene oxide platelets are embedded in a chitosan matrix	Cost effective; sustainable	Absorbent	Acid yellow (AY) and Acid blue (AB) removal	Ultrasound	87% efficiency after 8 cycles	[22]
Scallop shell	Fe_3_O_4_ NPs are coated on a shell surface	Efficient; low cost	Absorbent	Reactive black 5 (RB-5) removal		100% after 6 consecutive runs	[23]
Kaolinite/Chitosan	Composite beads with different ratios; self-assembly	Easy fabrication; eco-friendly	Chitosan has polycationic nature	Remazol red (RR)	pH		[24]
Kaolin	Kaolin is coated with Fe_3_O_4_ NPs	Easy separation by magnets	Adsorbent	Acid red 14 (AR-14)		Efficiency 88.43% after 6 cycles	[25]

**Table 2:** TiO_2_-based nanocomposites for photo-degradation of azo dyes.

Morphology	Improvements Over TiO_2_	Mechanisms	Usage	Light	Stability + Reusability	References
h-BN–TiO_2_	Band gap energy E_g_ = ~2.3–3.3 eV	h-BN (like graphene) accepts electrons and inhibits hole/electron (h^+^/e^−^) recombination	Orange II (OII); Reactive black 5 (RB-5)	UV Visible	k = 0.0762 min^−1^ (OII); k = 0.0164 min^−1^ (RB-5)	[26]
MgO nanoparticles within a TiO_2_ framework via surfactant-aided sol-gel approaches	Surfactant ensures uniform dispersion of the magnesium dopant onto titanium	High MgO content provides a high energy level for photo-degradation	Methyl orange (MO); Methylene blue (MB)	Visible	82.4% (MB); 77.8% (MO)	[27]
TiO_2_ was immobilized on the surface of fly ash, on which conductive polymer was grafted	Reduced band gap (E_g_ = 1.5–3.0 eV for conductive polymer) easy removal and reuse	Semiconductor produces ·OH and ·O_2_^−^ radicals	Reactive red 45 (RR-45)	Visible + Solar	Catalytic efficiency dropped quickly after the first run	[29]
N-(urea) and C–N (L-asparagine)-doped TiO_2_–CuO nanocomposite	TiO_2_ band gap was narrowed to the visible light region	CuO is a p-type semiconductor (E_g_ = 1.2–1.5 eV), and N-cap reduces h^+^/e^−^ recombination	Direct red 16 (DR-16)	Visible	k = 0.0403 min^−1^	[30]
N–Fe co-doped-TiO_2_/SiO_2_	Simultaneously remove Cr(VI) and azo dyes (using continuous flow photo reactor)	The non-metal anion dopants increase the percentage of the anatase phase of TiO_2_; SiO_2_ increases surface area	Cr(VI) Basic red 29 (BR-29); Basic blue 41 (BB-41); Basic yellow 51 (BY-51)	Visible or solar	91.73% (Cr^6+^) 85.64% (BR-29) 87.23% (BB-41); 58.59% (BY-51); 70% efficiency after the 7th 12 h cycle	[31]
Cu-doped TiO_2_–Bi_2_O_3_	Better efficiency using visible light	TiO_2_/Bi_2_O_3_ prevents recombination rates of photo-induced h^+^/e^−^ pairs; Cu reduce band gap to visible region	Methyl orange (MO)	LED lamp (λ > 410 nm)	79% efficiency after 5 cycles	[32]
Mn, Mo, and La/TiO_2_ activated carbon (AC)	High absorption ability in the visible light region; cost effective; easy removal	Metals reduce the recombination rates of hole/electron (h^+^/e^−^) pairs	Reactive red 198 (RR-198); textile wastewater	Visible	91% (RB-198) 52.78% efficiency after the 4th run	[33]
Fe^3+^-doped TiO_2_–bentonite clay	Remove total organic carbon (TOC)	Photo-Fenton reaction; nanocomposite reduces hole/electron (h^+^/e^−^) recombination rate	Orange II (OII)	254 nm UVC	100% decoloring	[34]
ZnO/TiO_2_ Phase transformations	ZnO absorbs a larger fraction of UV light than TiO_2_	Large surface area; hole can transfer from TiO_2_ valence band to ZnO valence band	Congo red (CR)	UVC lamp	discoloring after 10 min	[35]
Ferrites encapsulated with TiO_2_	Easy catalyst recovery	Larger specific area (43.2 m^2^/g) than TiO_2_ (31.1 m^2^/g) Dyes degrades in bulk solution by radicals	Textile effluents	UV (253.7 nm)	65% efficiency at pH = 7; 80% efficiency at pH = 12	[36]
Fe_3_O_4_/SiO_2_/TiO_2_	Fe_3_O_4_-core was coated with silica (SiO_2_), which was, in turn, coated with TiO_2_	Core Fe_3_O_4_ NPs SiO_2_—inner layer TiO_2_—outer layer	Methyl Orange (MO); Methylene blue (MB)	UV (365 nm)	90.2% (MO) 100% (MB); no efficiency decrease after 5 runs	[37]
TiO_2_–hydrogel	Good removal performance; pH sensitivity facilitates retrieval of catalyst at the end	TiO_2_ entrapped in the hydrogel	Acid orange 7 (AO-7); Acid red 88 (AR-88)	UV	98% AR-88 71% AO-7	[38]
TiO_2_ NPs/hydrogel	Hydrogel as supporting material for TiO_2_; easy removal	Hydrogel both supports TiO_2_ and absorbs pollutants	Acid red 18 (AR-18); Acid blue 113 (AB-113); Reactive black 5 (RB-5) Direct black-78	UV–sun-like illumination	85% efficiency after 3rd cycle	[39]
TiO_2_–SiO_2_ hollow nanospheres	Enhanced photocatalytic performance	Larger surface area (up to 1105 m^2^/g)	Methyl orange (MO); Phenol	UV light; sunlight	95% MO	[40]
TiO_2_–SiO_2_	Sol-gel methods; enhanced photocatalytic and thermal properties	Ti–O–Si bond and amorphous SiO_2_ increases the stability of anatase TiO_2_, increases surface area, and limits the growth of crystallites	Congo red (CR)	UVC lamp	98% CR	[41]
Au NPs/Preyssler acid/TiO_2_	Eco-friendly; no free Au contamination	Au-NPs increase charge separation between the excited electron (e^−^) and hole (h^+^); Preyssler is both a stabilizer and a reducing agent	Malachite green	UV	95% efficiency after the 3rd cycle	[42]
Sonic-TiO_2_/Bi_2_O_3_	More efficient than pure TiO_2_ and stirred TiO_2_/Bi_2_O_3_	High energy of ultrasonic wave promotes the crystallization of the TiO_2_ NPs and Bi_2_O_3_ NPs that grow on the surface to benefit the charge transfer	Orange II (OII)	Visible (λ > 400 nm)	94.7% OII	[43]
Graphene/TiO_2_–Ag	Extended light absorption range	Band gaps of nanocomposites are ~3.05–3.09 eV	Amaranth dye	UV; Sun	85.3–98% graphene/TiO_2_–Ag; 99% reduced graphene/TiO_2_–Ag	[44]
ZnO/TiO_2_ coated on stainless-steel electrode	Combined photoelectrocatalytic and electrochemical methods	ZnO/TiO_2_ composite reduced the band gap; electrical field separate photo-induced electron–hole pairs	Acid orange 7 (AO-7); Chemical (C1); chemicaloxygen demand (COD)	Solar irradiation	97% AO-7 99% COD	[45]

**Table 3:** Other nanocomposites for photo-degradation of azo dyes.

Morphology	Advantages	Mechanisms	Usage	Light	Stability + Reusability	References
Rice-like Ag/Al_2_O_3_	Good surface:volume ratio; good absorption; Capacity for Pb^2+^ (250 mg/g)	Surface area 241.219 m^2^/g	Pd^2+^; Acid violet 7 (AV-7)	Visible	86.7–100% AV-7; absorbs Pb^2+^ (215 mg/g)	[47]
Au NPs/(H_6_P_2_W_18_O_62_)	Au nanoparticles were incorporated in Wells–Dawson hetero-polyacid nanocomposite film	Heteropolyacids are powerful oxidants; reduced form is a strong reducing reagent and stabilizer	Methyl orange (MeO); Methylene read (MR)	UV 254 nm	96% MeO; 87.2% MR	[48]
CuBi_2_O_4_/Bi_3_ClO_4_	Strong visible light absorption; fine particle size; proper band gap; high charge separation efficiency	Hole (h^+^) and superoxide radical (·O_2_^−^) are prevailing active species	Acid brown 14 (AC-14)	Visible	92% efficiency (AC-14); 75% TOC removal	[49]
Bi_2_O_3_/Nexar polymer	Efficient; environmentally friendly; easy regeneration and reuse	Bi_2_O_3_ E_g_ = 2.1–2.8 eV; Nexar—membrane	Methyl orange (MO); Methylene blue (MB)	UV-vis or Blue	53% dyes membranes are processed and restored	[50]
NiO/Bi_2_O_3_/Bi_3_ClO_4_	Effective separation and transfer of the photoexcited electron–hole pairs	Photoexcited superoxide radicals and holes are prevailing active sites	Acid red 88 (AR-88)	Visible	89% AR-88; 95% efficiency after 4 cycles	[51]
Fe_3_O_4_-Pd/ pepper-extract	Magnetic separation; hydrothermal procedure (nanostructures have preferential and controlled shape and size)	Free electron gas of noble metal forms localized surface plasmon (LSP)	Acid black; Acid brown	UV	97% (Acid black); 85% (Acid brown); magnetic removal of catalyst	[52]
g-C_3_N_4_/Fe_3_O_4_/p-RuNP	Simple and convenient (efficiency, energy, and reusability), and facile separation from the reaction mixture	E_g_ = 2.76 eV ·OH; radical is responsible for the reactions	Congo red (CR); Chlorazol black (CB); Evans blue (EB); Reactive read (RR)	Visible; LED; Sunlight	85% (CR); 73% (CB); 65% (EB); 80%(RR); 65–85% efficiency; 95% efficiency after 5 cycles	[53]
CoFe_2_O_4_/SrTiO_3_ perovskite	Efficient, stable, and magnetic photocatalyst; easy separation from water	N:SrTiO_3_ E_g_ = 2.58 eV CoFe_2_O_4_ Eg = 1.76 eV	Acid brown; Acid black	Visible	Acid brown 44%; Acid black 99%	[54]
Fe_3_O_4_-microsphere/ polypyrrole-Ag	Core–shell morphology Fe_3_O_4_ as N-type semiconductor; Polypyrrole (PPY) as p-type semiconductor	The doped Ag improve transportation and separation of photo-induced charges on the surface interface; Band gaps are E_g_ = ~2.5 eV for nanocomposites of conducting polymer	Methyl orange (MO); Orange II (OII)	UV	86.2% MeO; 80.4% OII	[55]
Li_2_B_4_O_7_/LiBO_2_/Li_3_BO_3_ (LBO)	Strong photo absorption; high-efficiency charge carrier separation; uniform particle size; Appropriate band gap	Superoxide radicals (·O_2_^−^) and holes (h^+^) are the prevailing active species	Acid violet 7	UV	91% AV7; 70% TOC	[56]
Ni(OH)_2_ nanosheets/ZnO nanorods	Well-crystallized ZnO; poorly crystallized Ni(OH)_2_·0.75H_2_O; absorbance edge shifts to red	Interface charge transfer (IFCT) from conducting band of ZnO to Ni(II) heterojunction	Rhodamine B; CR; MB; MO	Visible	82.6–98.4% MB	[57]
ZrS_2_–ZnO	ZrS_2_ has E_g_ = 1.68 eV The coexistence of ZrS_2_ increases the absorbance of ZnO in the UV region	e^−^ in the conducting bands of ZrS_2_ and ZnO reduce O_2_ to ·O_2_^−^, h^+^ in valence band of ZnO oxidize H_2_O to ·OH	Naphthol blue black (NBB)	UV	98.5% Efficiency after 4th run	[58]

## Data Availability

Not applicable.
